# Analysis and Simulation of Forcing the Limits of Thermal Sensing for Microbolometers in CMOS–MEMS Technology

**DOI:** 10.3390/mi10110733

**Published:** 2019-10-29

**Authors:** Hasan Göktaş, Fikri Serdar Gökhan

**Affiliations:** 1 Department of Electrical and Electronic Engineering, Harran University, Şanlıurfa 63000, Turkey; 2 Department of Electrical and Electronic Engineering, Alanya Alaaddin Keykubat University, Kestel, Alanya, Antalya 07450, Turkey; fsgokhan@gmail.com

**Keywords:** microbolometer, infrared sensor, complementary metal-oxide semiconductor (CMOS), high sensitivity, temperature sensor, microresonator, MEMS, clamped–clamped beam, thermal detector

## Abstract

Room-temperature highly sensitive microbolometers are becoming very attractive in infrared (IR) sensing with the increase in demand for the internet of things (IOT), night vision, and medical imaging. Different techniques, such as building extremely small-scale devices (nanotubes, etc.) or using 2D materials, showed promising results in terms of high sensitivity with the cost of challenges in fabrication and low-noise readout circuit. Here, we propose a new and simple technique on the application of joule heating on a clamped–clamped beam without adding any complexity. It provides much better uniformity in temperature distribution in comparison to conventional joule heating, and this results in higher thermal stresses on fixed ends. This consequently brings around 60.5× improvement in the overall temperature sensitivity according to both theory and COMSOL (multiphysics solver). The sensitivity increased with the increase in the stiffness constant, and it was calculated as 134 N/m for a device with a 60.5× improvement. A considerable amount of decrease in the operation temperature (36× below 383 K and 47× below 428 K) was achieved via a new technique. That’s why the proposed solution can be used either to build highly reliable long-term devices or to increase the thermal sensitivity.

## 1. Introduction

MEMS/NEMS (Micro/Nano-Electro-Mechanical Systems) resonators got tremendous attention in the last decades, especially with the increase in the demand for the internet of things (IOT), biosensors, gas sensors, and infrared (IR) sensing applications (night vision, gas detection, medical imaging, etc.). Photon detectors [1,2] and microbolometers [3,4] are the two widely used and well-known competitors in building IR sensors. Photon detectors suffer from the requirement of cryogenic cooling, intrinsic noise, relatively high cost, fabrication complexity, and being bulky and expensive. On the other hand, room-temperature microbolometers not only eliminate all these problems, but can also easily be implemented in widely used CMOS (complementary metal-oxide semiconductor) processes [3]. The working principle of the microbolometer is based on the conversion of incident radiation into heat via a plasmonic absorber, and then conversion of this heat into an electrical signal via a temperature sensor. This electrical signal can be either resistance change (non-resonant) or frequency change (resonant type), depending on the device type. Resonant sensors [5,6,7], in contrast to non-resonant sensors [8], are the most popular because they offer significant advantages [6], such as high-quality factor of 1 million [9], ultra-low-noise measurement, and highly accurate measurement. The working principle of MEMS-type resonant-based thermal sensors is based on the resonance frequency shift, with respect to change in the temperature.

Many different techniques have been applied to increase the sensitivity of resonant-type microbolometers, where the thermal sensitivity strongly depends on the temperature coefficient of frequency (TCF). Extremely small devices [10,11] have higher sensitivity and a higher TCF, but they have challenges, such as difficulties in fabrication, high density integration, and low-noise readout circuit implementation. Using NEMS offered better sensitivity and TCF by achieving better thermal isolation [6,12,13], but sacrifices low stiffness constant. High quality factor [9,14,15] was achieved to detect a much smaller frequency shift and consequently enables higher thermal sensitivity and a higher TCF, but this method either requires vacuum environment or fabrication complexity or fabrication compatibility problem with CMOS process. Using phase change materials [16] in building cantilever-type resonators increased their thermal sensitivity and TCF, while bringing the fabrication challenges and CMOS compatibility problem. A fixed–fixed beam-type device was built [17] to drastically increase the TCF in comparison to other well-known resonant-type devices (cantilever, tuning fork, and free-free beam). The |TCF| of 4537 ppm/K was achieved in [5], and 19,500 ppm/K was achieved in [17], with fixed–fixed beam, where CMOS allowed high density integration and low-noise readout circuit. Later, this method was improved, and a possibility of 31× improvement, with a |TCF| of 2,178,946 ppm/K in thermal sensing, was demonstrated [7] by not only combining joule heating with ambient temperature change but also keeping the pull-in voltage as small as possible. However, nonuniform temperature distribution via joule heating prevented this approach from going down to smaller scale and consequently having higher thermal sensitivity due to relatively high thermal stresses.

We present a new type of joule heating technique in this work that allows much better temperature uniformity throughout the beam in comparison to conventional joule heating. In contrast to [7], here, joule heating is applied on sidebars attached on the fixed ends of the clamped–clamped beam. This uniform temperature profile not only increases the thermal stress on the fixed ends but also allows building design with relatively higher stiffness constant, while keeping the temperature limit around 530 K [18]. This increase in return results in the increase in the sensitivity multiplier and TCF. The sensitivity multiplier was computed as 60.5×, with a 30 µm long and 0.9 µm thick device, according to both COMSOL and theory in comparison to the possibility of 31× demonstrated in [7]. The |TCF| for 60.8× improvement was calculated as 3,991,168 ppm/K in comparison to 2,178,946 ppm/K in [7], 19,500 ppm/K in [17], 4537 ppm/K in [5], 30 ppm/K in [6], 548 ppm/K in [10], 86.2 ppm/K in [19], and 29.4 ppm/K in [13]. Furthermore, this uniform heating allows the design to have the same thermal sensitivity, with a conventional joule heating scheme [7], while drastically decreasing the operation temperature and enabling long-term operation. In other words, the proposed design methodology can be used either to increase the thermal sensitivity or to decrease the operation temperature for high reliability.

Using aluminum or composite structure in building a clamped–clamped beam in a CMOS 0.6 process showed almost no difference in the sensitivity multiplier, even at different sizes, where the stiffness constant stayed the same. That’s why optimum design with a critical temperature below 530 K can be designed in any fabrication process/technology (CMOS, silicon, Silicon on insulator (SOI), etc.), regardless of the material and size, as long as the stiffness constant is limited to 134 N/m.

## 2. Theory, Design, and Optimization

The main mechanism behind the wide-range frequency tuning is the thermal stresses created via joule heating, as demonstrated for the first time in [17]. Another study [7] conducted on CMOS clamped–clamped beam proved that thermal stress is the dominant factor on the temperature sensitivity, and the stresses caused by the increase in the pull-in force adversely affect the sensitivity. The main goal of this study is to increase the temperature uniformity throughout the beam to get further increase in the thermal stress at a relatively low temperature, and consequently get higher thermal sensitivity for microbolometer applications.

### 2.1. Design for Uniform Heating

**Figure 1:**
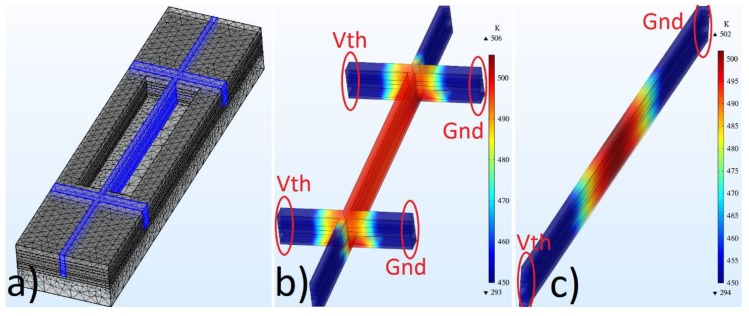
(**a**) UniJoule structure built via a CMOS process for performance improvement, and temperature distribution as a result of joule heating application of (**b**) UniJoule structure and (**c**) conventional clamped–clamped beam computed via COMSOL.

One of the main disadvantages of joule heating is the well-known nonuniform temperature distribution throughout the conventional clamped–clamped beam [17] (Figure 1c). Its temperature profile can be derived by combining thermal conduction and heat generation equations:

(1) $$T_{a}-\;T_{b}=\;\frac{I^{2}\rho L^{3}}{2VAk}$$

where $T_{a}$ is the maximum temperature at the center of the beam, $T_{b}$ is the minimum temperature at the fixed ends, *I* is the current flow, *L* is the length, ρ is the density, *V* is the volume, *A* is the cross-sectional area, and *k* is the thermal conduction constant.

The conventional clamped–clamped beam was converted into a new type of structure (called UniJoule (Figure 1b)) by adding sidebars for the sake of better temperature uniformity. The design was built via a CMOS 0.6 um process, and all the details related to the fabrication process were given in [7,17], where CHF_3_/O_2_ was used to etch SiO_2_, and XeF_2_ was used to etch silicon underneath the beams. Here, the voltage (Vth) and ground (Gnd) applied on the sidebars (Figure 1b) in contrast to the conventional one, where Vth and Gnd applied on the fixed ends (Figure 1c). This enables the maximum temperature locating on fixed ends of the clamped–clamped beam embedded in the UniJoule structure (Figure 1b) and consequently creates a much more uniform temperature distribution.

The COMSOL was used to model and simulate the entire 3-layer composite beam (Figure 1a), including the substrate layer, where the first layer is SiO_2_, second layer is Polysilicon, and third layer is Aluminum in the 3-layer composite beam. There are two silicon layers in the structure: the first one is the etched one to allow the beam to resonate, and the second one is the substrate that carries all the layers. The substrate thickness was kept small to decrease the simulation time, while the thermal conduction constant of the substrate was recalculated according to thermal resistance (*R* = *H/k*, *H* = substrate thickness, *k* = thermal conductivity) for the sake of the accuracy. The substrate bottom was kept as a fixed surface in solid mechanics, and its temperature was kept at 293 K in a heat-transfer module. The conductivity of polysilicon was set to 1.16 ×$\;10^{5}$ S/m [17], and the fine mesh with a tetrahedral structure was used to complete the UniJoule structure in COMSOL. Here, an electric current model was used to apply joule heating, a heat transfer module was used to calculate temperature distribution throughout the beam, and a solid mechanics module was used to calculate deflection, mode shapes, and resonance frequency, with respect to temperature. The high-density sweep points (up to 2.3 ×$\;10^{-6}$ V resolution) in the application of joule heating (Vth) were applied to achieve high accuracy in the results. The convective cooling was added to the heat-transfer module for the sake of high accuracy.

### 2.2. Design for High Thermal Sensitivity

**Figure 2:**
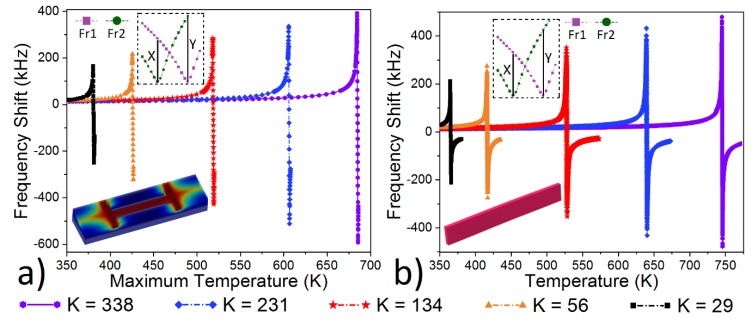
Frequency shift (FS = Fr1 − Fr2) with respect to 1 Kelvin change by (**a**) COMSOL for UniJoule structure with Vth application and (**b**) Equation (2) for uniformly heated conventional clamped–clamped beam, when stiffness constant (K) changed from 29 to 338 N/m; here, X represents positive max, and Y represents negative max of FS [22] in inset.

The detailed study was conducted on the thermal sensitivity of the UniJoule structure (Figure 2a) via COMSOL, and the results were compared to the uniform heating case (Figure 2b), where the sensitivity was derived via the use of resonance frequency, with respect to axial load [20]:

(2) $$f=\;\frac{4.73^{2}}{2\pi L^{2}}\;{\left(1+\frac{PL^{2}}{EI\pi^{2}}\right)}^{\frac{1}{2}}{\left(\frac{EI}{m}\right)}^{\frac{1}{2}}$$

(3) $$P=\frac{4}{h}\left(E_{1}I_{1}+E_{2}I_{2}\right)\left(\frac{(\alpha_{1}-\;\alpha_{2})T\;}{\frac{h}{2}+\left(\frac{2E_{1}I_{1}+E_{2}I_{2}}{hb}\right)\left(\frac{1}{E_{1}t_{1}}+\frac{1}{E_{2}t_{2}}\right)}\right)$$

where *I* is the moment of inertia, *m* (kg/m) is the mass per unit length, *P* is the total compressive axial load [21], *L* (m) is the length, *h* (m) is the width and *b* (m) is the thickness, and *E* is the Elastic modulus of the 2-layer composite beam in Equation (2). In Equation (3), $t_{1}$ is the width, $\alpha_{1}$ is the thermal expansion constant, $E_{1}$is the Elastic modulus, and $I_{1}$ is the moment of inertia for the aluminum layer, $t_{2}$ is the width, $\alpha_{2}$ is the thermal expansion constant, $E_{2}\;$is the Elastic modulus, and $I_{2}$ is the moment of inertia for SiO_2_ layer. Here, the 3-layer composite beam (Figure 1) was converted into an equivalent 2-layer composite beam via COMSOL, to find the total compressive axial load in Equation (3), where the first layer is SiO_2_ and the second layer is Aluminum. The Equation (2) was verified and matched with the measurement results in [18], while the increase in the thermal sensitivity with the increase in the joule heating application was verified with measurements in [17,18]. Further studies [7] demonstrated that the decrease in the pull-in force around the beam-bending point increased the thermal sensitivity. However, one of the main problems and limitations for this type of device is the maximum allowable thermal stress and, consequently, the maximum allowable temperature that the resonator can tolerate. This was measured and verified around 530 K for the same type of structure in the CMOS process [18]. The possibility of a 31× improvement in thermal sensitivity was demonstrated in [7] for the conventional 57 µm long clamped–clamped beam with a maximum temperature of 530 K, around the beam bending point. In contrast to [7], here we built a UniJoule structure that allows uniform heating throughout the structure. A 60.8× ((284 + |−428|) kHz/11.7 kHz) improvement was achieved with a 30 µm long device, according to COMSOL (Figure 2a), and it was calculated around 60.5×, according to Equation (2) (Figure 2b), where the temperature (at the beam bending point [7]) was 520 K, according to COMSOL, and 529 K, according to Equation (2). This improvement is attributed to the fact that uniform heating allows for a larger stiffness constant, while keeping the critical temperature below 530 K, according to both COMSOL and Equation (2), and it consequently results in higher thermal sensitivity.

The Frequency shift (FS) was calculated by taking the difference between two resonance frequencies Fr1 and Fr2 (more details given in [7]), where the ambient temperatures were set as 293 and 294 K, respectively. The sensitivity multiplier (total maximum FS/minimum FS) was increased from 37 to 82, according to COMSOL, and from 36 to 82, according to Equation (2), when the stiffness constant increased from 29 to 338 N/m (Figure 2). The optimum structure was selected to be a 30 µm long beam, where the sensitivity multiplier is around 60.5×, with a stiffness constant of 134 N/m, according to both COMSOL and theory. A decent uniformity in temperature distribution for all UniJoule structures (Figure 2a) was achieved where the difference between the maximum and the minimum temperature throughout the beam is less than 6.5%.

**Figure 3:**
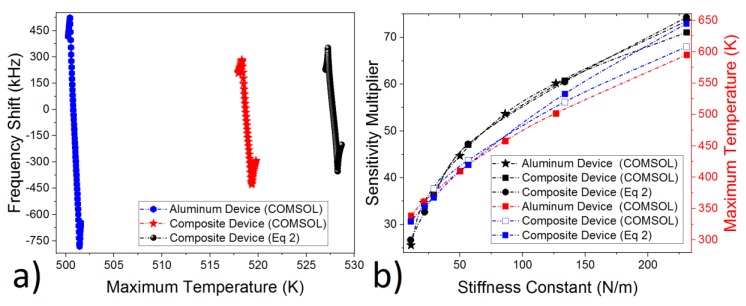
(**a**) Frequency shift with respect to temperature and (**b**) the relationship between stiffness constant, sensitivity multiplier, and maximum allowable temperature for both aluminum and composite structure (Figure 1) derived via COMSOL and Equation (2).

More studies were conducted on the effect of the stiffness constant and material configuration on the sensitivity multiplier and maximum allowable temperature (Figure 3). The first design was built on a composite structure (Figure 1) [5], while the second one was built by only using an aluminum layer [22] in the CMOS process. The results suggest that the sensitivity multiplier depends on the stiffness constant, rather than the material type used in building the resonator. It is around 60.5×, with a temperature below 530 K, for the composite structure (according to both COMSOL and theory), and it is 60.2× at 502 K for the aluminum resonator, where the stiffness constant is 127 N/m for the aluminum design and 134 N/m for composite structure (Figure 3a). Although the sensitivity multiplier is around 60× for both aluminum and the composite structure, the aluminum resonator still shows overall better thermal sensitivity (total |FS_aluminum_| = 1306 vs. total |FS_composite_| = 712) (Figure 3a). This is attributed the fact that aluminum has a larger thermal expansion constant ($\alpha_{Aluminum}$ = 23.1 ×$\;10^{-6}$ 1/K) in comparison to composite structure, where SiO_2_ ($\alpha_{\mathrm{SiO}2}\;$= 0.5 ×$\;10^{-6}$ 1/K) and Polysilicon ($\alpha_{Polysilicon}$ = 2.65 ×$\;10^{-6}$ 1/K) decreases the overall thermal expansion constant.

The relationship between the stiffness constant, the sensitivity multiplier, and the maximum temperature around the beam bending point was analyzed. Both the aluminum and composite structure had a relatively sharp increase in the sensitivity multiplier, where the stiffness constant was smaller than 50 (Figure 3b). The sensitivity multiplier and the maximum temperature increases with the increase in the stiffness constant. The maximum allowable temperature was set to 530 K, and that’s why the maximum stiffness constant was calculated as 134 N/m, with a maximum sensitivity multiplier of 60.5×. Moreover, this method can be also used to drastically decrease the operation temperature, while keeping the sensitivity multiplier relatively high. The sensitivity multiplier around 36×, with a temperature below 383 K, and 47×, with a temperature below 428 K, can be achieved according to both COMSOL and the theory (Figure 2 and Figure 3). This feature is especially important for long-term reliable operations.

This finding suggests that using different material or different fabrication processes in building a resonator doesn’t affect the sensitivity multiplier or the maximum temperature around beam bending at all (Figure 3). The only critical parameter setting the maximum sensitivity multiplier is the stiffness constant.

### 2.3. Design for Relatively Low Power Consumption

**Figure 4:**
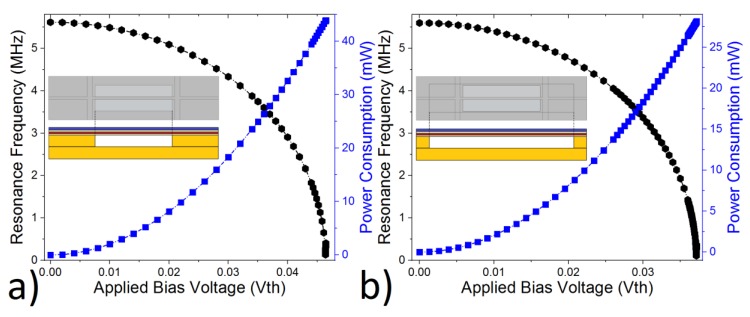
The power consumption and bending voltage for a UniJoule structure (Figure 1b), where (**a**) there is no isotropic on fixed-ends and (**b**) there is a 13 µm isotropic etching on both fixed-ends.

The power consumption is another critical parameter for the sensor designs, especially with the increase in demand in the internet of things (IOT) applications. The XeF_2_ process for isotropic etching of silicon layer is a well-known and widely used process, especially in CMOS–MEMS [5,17]. Here, we applied isotropic etching on the clamped–clamped beam (Figure 1b) for the sake of better thermal isolation. Design-1 has a smaller mask opening (Figure 4a inset), while design-2 has a wider mask opening (Figure 4b inset) to allow 13 µm silicon etching on both sides. This etching process decreases the thermal conduction from beam to substrate, and this allows better thermal isolation. This, in return, enables less power consumption to generate the same amount of heat on the beam, according to Equation (1). Thanks to high thermal conductivity of the silicon layer (1.3 W${\mathrm{cm}}^{-1}{}^{\circ}C^{-1}$), this technique would allow a noticeable decrease in the power consumption.

Both design-1 and design-2 were heated via applied bias voltage (Vth) (Figure 1b), till the devices reached the bending point, where the maximum thermal sensitivity was achieved (Figure 2) [7]. The bending point was reached at Vth = 0.0464 V for design-1 and Vth = 0.0372 V for design-2. This represents around 43.8 mW power consumption for design-1 and 28 mW for design-2. In other words, the power consumption was decreased by 36% via using a wider mask with the XeF_2_ isotropic silicon etching process.

The effect of isotropic etching was also investigated on the thermal-sensitivity performance. Although the etching process resulted in lower power consumption, both design-1 and design-2 showed the same sensitivity multiplier. This is attributed to the fact that both designs have the same stiffness constant (Figure 3). There is only a slight difference between the two designs. Design-1 reached the bending point around 527 K, while design-2 reached it around 519 K.

## 3. Conclusions

A new type of joule heating scheme was demonstrated for the sake of uniform temperature distribution throughout the beam. This, in return, enabled the higher thermal stresses and higher sensitivity multiplier. Around a 60.5× improvement in thermal sensing was achieved and verified via both COMSOL and theory, while keeping the device temperature below 530 K. The very same method can also be used to drastically decrease the operation temperature and consequently enables long-term reliable operation, where 36× with a temperature below 383 K and 47× with a temperature below 428K was achieved according to both COMSOL and theory. The results suggest that the maximum sensitivity multiplier can be achieved when the stiffness constant is around 134 N/m, regardless of the materials or process used in building the devices. The |TCF| was calculated around 3,991,168 ppm/K, where the applied bias voltage (Vth) is 0.0372 V. It should be noted that this improvement (60.5×) in the thermal sensitivity was achieved without any need for a complex and expensive fabrication process or even special layers, such as 2D materials. This would be very crucial and helpful in supporting the studies conducted on medical imaging. Further improvement was achieved via isotropic etching applied on silicon layer. The power consumption decreased from 43.8 to 28 mW with the decrease in the thermal conduction according to COMSOL. This can be very beneficial for applications that requires compact size, low cost, and wireless communications, such as the internet of things (IOT).
